# Selective Cloud Point Extraction for the Spectrophotometric Determination of Cetylpyridinium Chloride in Pharmaceutical Formulations 

**Published:** 2013

**Authors:** Ali Reza Zarei, Hayedeh Bagheri Sadeghi, Samira Abedin

**Affiliations:** a *Faculty of Chemistry and Chemical Engineering, Malek Ashtar University of Technology, Tehran, 158751774-, Iran. *; b *Department of Chemistry, Islamic Azad University, Tehran Central Branch, Tehran, Iran. *

**Keywords:** Cloud point extraction, Cetylpyridinium chloride, Spectrophotometry, Pharmaceutical products

## Abstract

In this work, we developed a simple and selective method for separation and spectrophotometric determination of trace amounts of cetylpyridinium chloride (CPC) in pharmaceutical products using cloud point extraction (CPE) technique. The method is based on cloud point extraction of the CPC in alkali conditions using of nonionic surfactant Triton X-114. Under optimal conditions, the calibration graph was linear in the range of 0.50-30 μg/ mL of CPC with r=0.9993 (n=10). Average recoveries for spiked samples were determined to be between 95–104%. The relative standard deviation (RSD) for 5.0 μg/mL of CPC was 1.86 % (n=10). Also, the use of micellar extraction for extracting CPC was enhanced the molar absorptivity (ε) from 1.83×10^3^ L/mol.cm in aqueous solution to 1.539×10^4 ^L/mol.cm in surfactant-rich phase. The proposed method was applied for the determination of CPC in a commercial mouth washer product

## Introduction

**Scheme 1:**
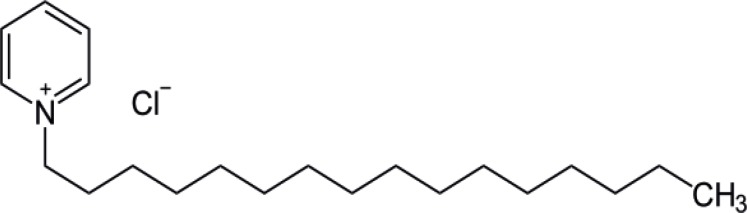
The chemical structure of cetylpyridinium chloride (CPC

Cetylpyridinium chloride (CPC) is a quaternary ammonium compound (QAC) (Scheme 1), which can be classified as a heteroaromatic ammonium salt, that has one long-chain alkyl group and the remaining is an aromatic system such that the quaternary nitrogen is part of an aromatic system like pyridine in the case of CPC (1). This compound is the active chemical and is widely used in industrial and pharmaceutical products, especially in manufacturing of mouth washer, drupes and cosmetics (2, 3). The antimicrobial activity is due to the interaction of basic cetylpyridinium ions with acidic molecules on bacteria, which subsequently inhibits bacterial metabolism by forming weak ionic compounds that interfere with bacterial respiration (4, 5). According to the Food and Drug Administration (FDA) Dental Plaque Subcommittee, cetylpyridinium chloride has been accepted as a safe and effective antimicrobial for the treatment of plaque-induced gingivitis (6). Research in oral bacteriology has indicated that 0.05 to 0.5% CPC found in mouthwashes reduces or inhibits bacterial gingivitis, biofilm, and plaque formation (7, 8). 

Different methods including potentiometry (9, 10), voltametry (11), gas chromatography (12), high performance liquid chromatography (13), capillary electrophoresis (14, 15), chemiluminescence (16), and spectrophotometry (17-19) have been reported for the determination of CPC. Among these methods, ion-pair spectrophotometric methods using anionic dyes as counter ions have been widely used for the determination of cationic surfactants, due to simplicity, rapidity and cost-effective (20-22). Generally, in these analytical methods, separation of ion pair product is carried out using conventional liquid-liquid extraction which is a time consuming and multistage operation and have used large amounts of potentially toxic organic solvents which are often hazardous and expensive.

In the last decade, increasing interest on the use of aqueous micellar solution has been found in the field of separation science (23, 24). CPE is a suitable alternative separation and preconcentration technique, which is based on the phase behavior of nonionic and zwitter ionic surfactants in aqueous solutions which exhibit phase separation after an increase in temperature or the addition of a salting-out agent (25). Generally, above a certain temperature, a single phase non-ionic surfactant micelle aqueous solution separates into a dilute aqueous phase and a surfactant-rich phase. Any analyte solubilized in the hydrophobic core of the micelles, will separate in the small volume of the surfactant-rich phase. CPE is in agreement with the principles of the green chemistry (26). It is a green method for the following reasons: (i) it uses from diluted solution of the surfactants that are inexpensive and low cost, and (ii) surfactants are not toxic, not volatile, and easily flammable. This simple technique enables us to avoid hazardous organic solvents and allows to achieve a much higher concentration of analyte than in the case of liquid-liquid extraction, because the micellar phase volume is about 10-100-fold less than the volume of an aqueous phase (27, 28). The effectiveness of the cloud point extraction is due to its high selectivity and the possibility of obtaining high coefficients of preconcentration while analyzing small sample volumes. Cloud point extraction has been widely used for the separation, purification and preconcentration of a variety of pharmaceutical products (29-33). 

This work is mainly focused on the suitability of CPE combined with UV-Vis spectrophotometry for determination of CPC. The influence of the different experimental parameters on the reaction and extraction steps were discussed. To evaluate the applicability of the proposed method, it was then applied for the determination of CPC in mouth rinse formulations.

## Experimental

*Apparatus*

A Hitachi model 3310 UV-Vis spectrophotometer with 1-cm quartz cells (1.0 mL) was used for recording absorbance spectra. A centrifuge with 10 mL calibrated centrifuge tubes (Hettich, Germany) was used to accelerate the phase separation process.

*Reagents*

All chemicals and reagents are analytical reagent grade, and distilled water was used throughout the experiments. A stock solution of 1000 μg/mL of CPC was prepared by dissolving 0.100 g cetylpyridinium chloride (Merck) in water and diluting to 100 mL in a volumetric flask. A 5 M NaOH was obtained by dissolving 20 g sodium hydroxide (Merck) in water and diluting to 100 mL in a volumetric flask. 2.0 g of Triton X-114 was dissolved in 100 mL of distilled water to give a 2.0 % (w/v) of surfactant solution.

*Recommended procedure*

For the cloud point extraction under optimum conditions, an aliquot of the CPC solution so that its final concentration would be in the range of 0.50-30 μg/mL, 1.0 mL of 5 M NaOH solution were transferred into a 10 ml screw cap glass test tube with conical bottom. The solution was diluted to approximately 8 mL with distilled water and left to stand in a thermostat bath at 50 ^0^C for 5 min. Afterwards, the solution was cooled to room temperature. Then, 1.0 mL of 2.0 % (w/v) of Triton X-114 solution was added and the final volume was completed to the mark with distilled water. The solution was shaken for 30 sec and separation of the aqueous and surfactant-rich phase was accomplished by centrifugation for 3 min at 3800 rpm. The mixture was cooled in an ice-salt bath to increase the viscosity of the surfactant-rich phase, and the aqueous phase was easily decanted. The surfactant rich phase of this procedure was dissolved and diluted to 1.0 mL with ethanol and transferred to a 1.0-mL quartz cell for absorbance measurement at 347 nm against a blank solution. A blank solution was also run using water instead of CPC. 

## Results and Discussion

**Figure 1:**
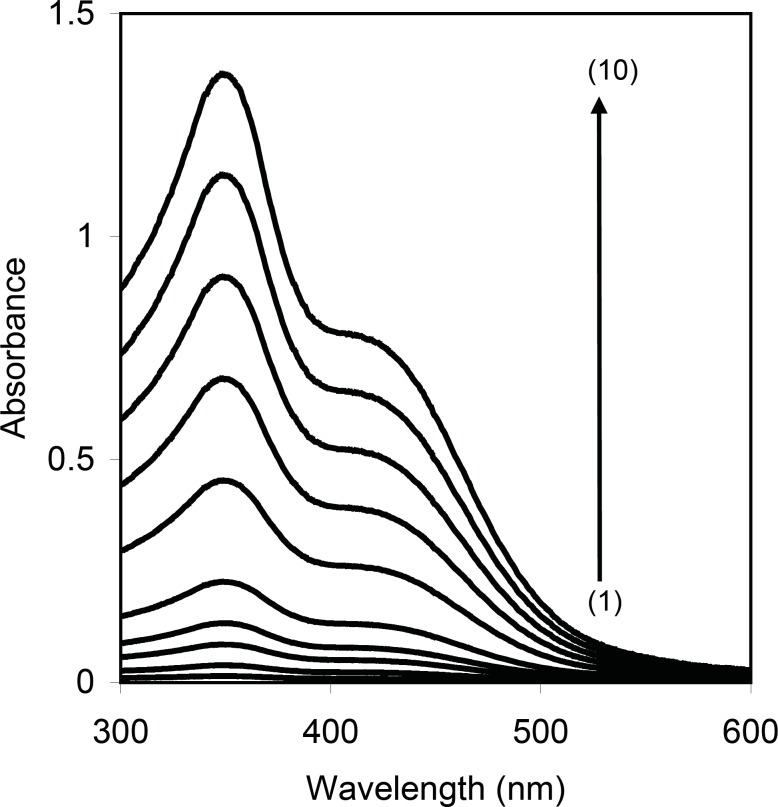
Absorption spectra of CPC after CPE, Conditions: CPC, (1) 0.50 (2) 1.0 (3) 2.0 (4) 3.0 (5) 5.0 (6) 10 (7) 15 (8) 20 (9) 25 (10) 30 μg/mL; NaOH, 0.5 M; T= 50 0C; t=5 min; Triton X-114, 0.20 % (w/v).

Cetylpyridinium chloride is a cationic surfactant which it is converted to non ionic form (yellow color) in alkali media and can be extracted into non-ionic surfactant, Triton X-114. Therefore, it can be a suitable method for separation of CPC by CPE method. Figure 1 shows that the absorption spectrum of the CPC in surfactant-rich phase exhibits a maximum absorbance at 347 nm. Therefore, all absorbance measurements were performed at this wavelength. For achieving the highest efficiency and sensitivity, the influence of effective variables was investigated and optimum conditions were obtained. 

*Effect of the sodium hydroxide concentration*

**Figure 2:**
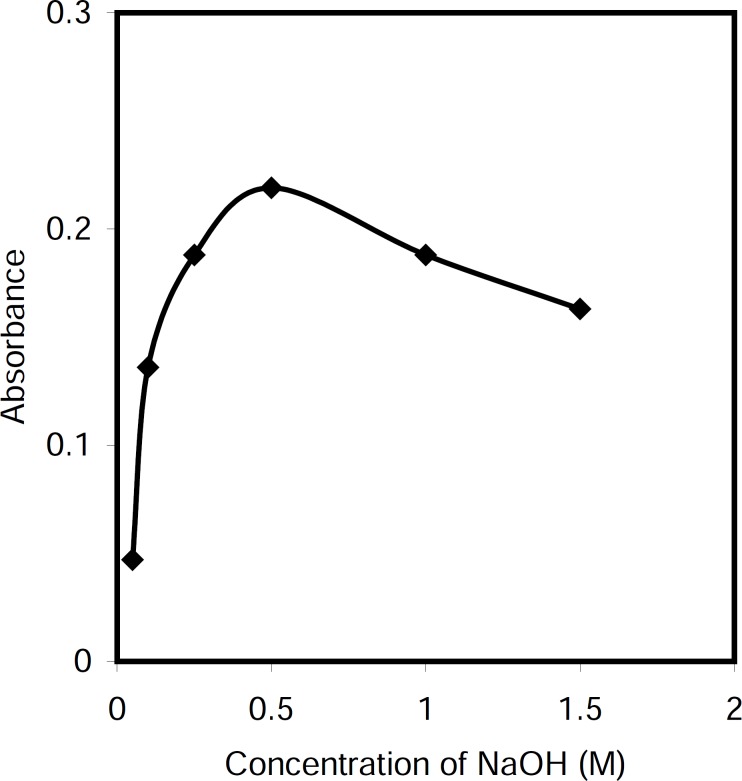
Effect of sodium hydroxide concentration on the absorbance of the system, Conditions: CPC, 5.0 μg/mL; T= 50 °C, t=5 min; Triton X-114, 0.20 % (w/v).

Cetylpyridinium chloride is a cationic surfactant which it is converted to color non ionic form in alkali media. Therefore, the effect of sodium hydroxide concentration on the absorbance of the system was investigated within the range 0.05-1.5 M. The results revealed that the absorbance increased by increasing reagent concentration up to 0.5 M, and decreased at higher concentrations (Figure 2). Therefore, a concentration of 0.5 M sodium hydroxide was applied in the proposed method. 

*Effect of temperature*

**Figure 3:**
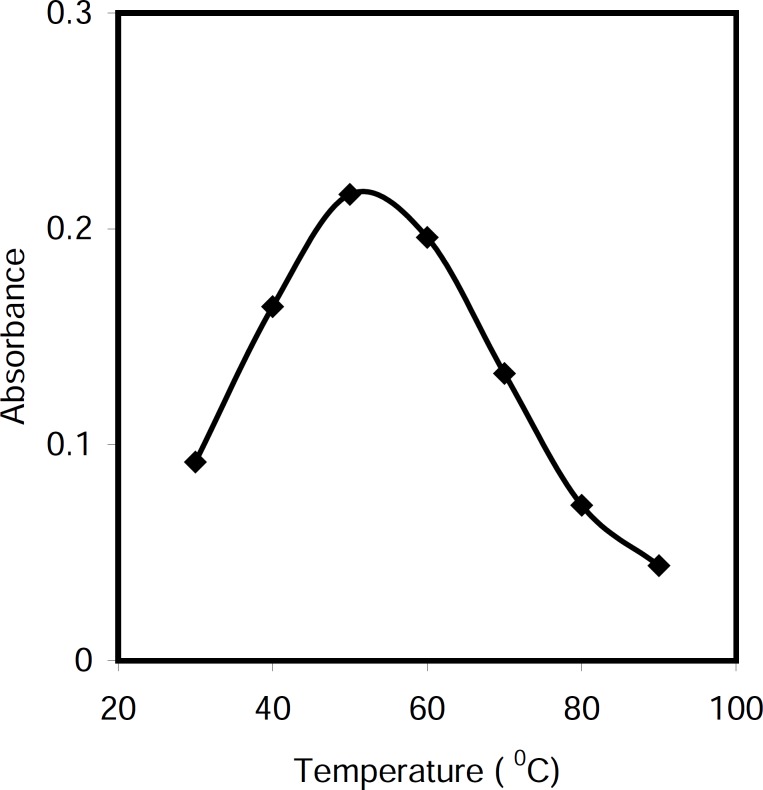
Effect of temperature on the analytical signals, Conditions: CPC, 5.0 μg/mL; NaOH, 0.5 M; t = 5 min; Triton X-114, 0.20 % (w/v).

The effect of temperature on the conversion of cetylpyridinium chloride to nonionic form in alkali media was studied in the range 30-80 °C. As can be seen in Figure 3, the maximum absorbance was achieved up to 50 °C and decreased at higher temperature. Therefore, the reaction was carried out into water bath at 50 °C. 

*Effect of time of reaction*

**Figure 4:**
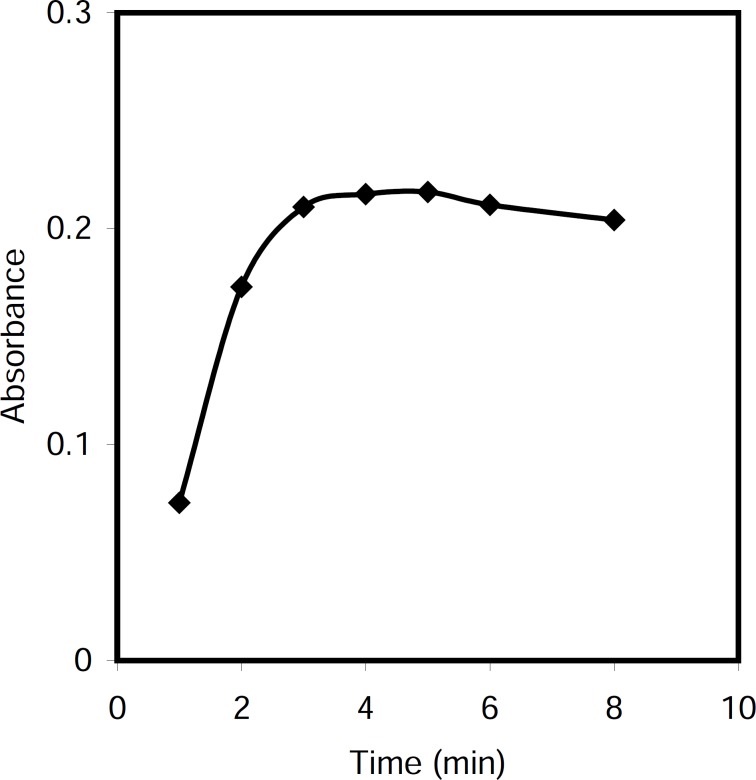
Effect of time of reaction on the analytical signals, Conditions: CPC, 5.0 μg/mL; NaOH, 0.5 M; T= 50 ^0^C, Triton X-114, 0.20 % (w/v).

The effect of time on the formation of nonionic CPC in alkali media was investigated in the range 1-10 min. It was found that reaction was completed in about 5 min at 50 °C (Figure 4). 

*Effect of Triton X-114 concentration*

**Figure 5:**
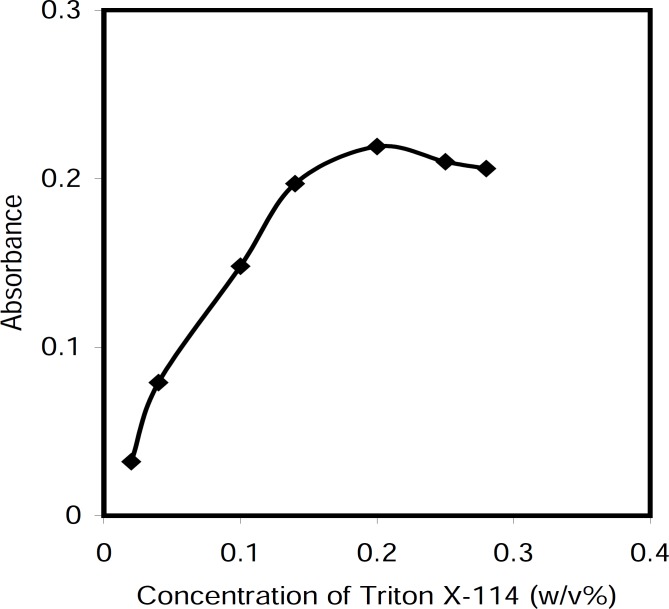
Effect of Triton X-114 concentration on the absorbance of the system, Conditions: CPC, 5.0 μg/mL; NaOH, 0.5 M; T= 50 °C; t=5 min

The concentration of the surfactant used for CPE is an important factor. Preliminary investigations showed that the cetylpyridinium chloride nonionic form was completely extracted in Triton X-114. To obtain the optimal concentration of Triton X-114, the effect of its concentration was investigated on the absorbance of the extracted phase. The results illustrated in Figure 5 reveal that at the surfactant concentration of 0.20 % (w/v), more extraction occurred. This value was selected as the optimal concentration value.

*Effect of centrifugation time *

The centrifugation time does not have a considerable effect on the analytical characteristics of the CPE method. This parameter was examined in the range of 2-10 min at 3800 rpm. A time of 3 min was selected as optimum, since complete phase separation occurs in this time and no appreciable improvements were observed for larger times. 

*Analytical figures of merit*

Table 1 summarizes the analytical characteristics of the optimized method, including regression equation, linear range, molar absorptivity, correlation coefficient, limits of detection, enrichment factor and extraction recovery. Also, the improvnet factor that is defined as the ratio of the slope of the calibration graph for the CPE method to the slope of the calibration graph before extraction, was obtained 10.53. 

The enrichment factor (*EF*) was defined the ratio between the analyte concentration in the surfactant-rich phase (*C*_S_) and the initial concentration of the analyte (*C*_0_) in the aqueous sample (35). 

*EF= C*_S_ */ C*_0_ (1) 

The extraction recovery (*R%*) was defined as the percentage of the total analyte which was extracted in the surfactant-rich phase. 

*R%= EF*×*(V*_S_*/V*_a_q*)*×*100 *( 2 ) 

Where *R%*, *V*_S_*, *and *V*_aq_ are the extraction recovery, the volume of the surfactant-rich phase, and the volume of the aqueous sample, respectively. In order to examine the enrichment factor, three replicate extractions were perfomed at optimal conditions from aqueous solutions consisting 10 μg/mL of CPC. *C*_S_ was calculated from the calibration graph obtained by direct spectrophotometric determination (Table 1, before extraction, A= 0.0043C-0.0051). Based on Eqs. (1) and (2), the enrichment factor and extraction recovery (*R%*) were obtained 9.88 and 98.83 %, respectively. 

**Table 1:** Analytical characteristics of the proposed method

*λ *_max_ (nm)	347
Regression equation after CPE (*n *= 10)	A = 0.0453C - 0.0099, R^2^ = 0.9986
Regression equation (*n*=10) before extraction	A = 0.0043C - 0.0051, R^2^ = 0.999
Linear range (μg/mL)	0.5-30 (10-300)a
Molar absorptivity (L/mol.cm)	1.539×104 (1.83×103)a
Limit of detection _b_ (μg/mL)	0.30
Reproducibility (R.S.D., %)	1.86
Improvenet factor	10.53
Enrichment factor (EF)	9.88
Extraction recovery (R%)	98.83

^a^ For before extraction.

^b^ Defined as *CL=3SB/m *where *CL, SB*, and m are the limit of detection, standard deviation of the blank, and slope of the calibration graph, respectively (34).

*Interference study*

To study the selectivity of the proposed method, the effect of various species on the determination of CPC was tested under the optimum conditions. For this purpose, sample solutions containing 5.0 μg/mL of CPC and different concentration of various species were prepared and the developed procedure was applied. The tolerance limit was defined as the concentration of added species that caused a relative error of less than ±5%. Similar surfactants as cetyltrimethylammonium bromide (CTAB) and cetyltrimethylammonium chloride (CTAC) were also tolerable to concentration of 100 time ratio (Table 2). This shows the good selectivity of the proposed method respect to extractive ion-pair spectrophotometric methods. 

**Table 2:** Tolerance limit of interfering species in the determination of 5.0 μg/mL of CPC

Species	Tolerane ratio (wspecies/wCPC)
**Cations and Anions: **Na^+^, K^+^, Mg^2+^, NH^4+^, Ba^2+^, Zn^2+^, Cd^2+^, Ca^2+^, Fe^3+^, Fe^2+^, SO_4_^2-^, NO^3-^, Cl-, , NO_2_-, Br-, F_-_, CO_3_^2-^, PO_4_^3-^, SCN-, Tartarate, Citrate	200:1
**Surfactants: **Cetyltrimethylammonium bromide (CTAB), Cetyltrimethylammonium chloride (CTAC), Sodium dodectyl sulfate (SDS), Triton X-100, Brij-35	*100:1 *

*Analytical application*

In order to validate the methodology, the proposed method was applied to the determination of CPC in commercial mouth washer products (Aquafresh, UK; Colgate, India; Oral-B, UK). A 5.0 mL of commercial mouth washer was transferred into 100 mL volumetric flask and diluted to the mark with distilled water. Then 2.0 mL aliquot was subjected to the CPE methodology as described in section 2.3. The results are summarized in Table 3. As can be seen, the recoveries corresponding to the additions of different concentrations of CPC to the samples were in the range 96.4-105.8%. Also, there is a good agreement between the results of the proposed method and the reference value of CPC content (500 μg/mL) of the mouth washer formulation. The results had excellent agreement with the reference value by performing t-test at the 95% confidence level (36). The results indicate that the proposed method is helpful for the determination of CPC in commercial mouth rinse formulations.

**Table 3:** Determination of CPC in commercial mouth washer pharmaceutical by proposed method

**Mouth washer pharmaceutical**	**CPC (μg/mL)**			
	**Added**	**Found** **a**	**Recovery (%)**	**Original sample** **b ** **(μg/mL)**
Aquafresh	0.0	5.10 ± 0.89	102c	510± 2.05
	5.0	9.92 ± 0.78	96.4	
	10	15.5 ± 0.69	104	
	20	25.7 ± 0.55	103	
Colgate	0.0	4.88 ± 0.86	97.6c	488± 1.89
	5.0	10.1 ± 0.83	104.4	
	10	14.7 ± 0.81	98.2	
	20	25.6 ± 0.67	103.6	
Oral-B	0.0	4.91± 0.93	98.2c	491± 1.76
	5.0	10.2 ± 0.86	105.8	
	10	15.3 ± 0.99	103.9	
	20	24.2 ± 0.77	96.5	

^a^ Average of three determinations± standard deviation.

^b^ Obtained amounts for mouth washers by proposed method.

^ c^ It was calculated by dividing by found amounts on original value after dilution (5.0 μg/mL).

## Conclusions

The new proposed procedure gives a highly selective and low-cost spectrophotometric procedure for determination of CPC. A nonionic surfactant has been used for separation of CPC and thus toxic solvent extraction has been avoided. Finally, the coupling of CPE with UV-Vis spectrophotometry, gave a very rapid and low-cost procedure for determination of CPC without requiring to sophisticated instruments such as HPLC and capillary electrophoresis.

## References

[1] Cutter CN, Dorsa WJ, Handie A, Rodriguez-Morales S, Zhou X, Breen PJ, Compadre CM (2000). Antimicrobial activity of cetylpyridinium chloride washes against pathogenic bacteria on beef surfaces. J. Food. Prot.

[2] Breen PJ, Compadre CM, Fifer EK, Salari H, Serbus DC, Lattin DL (1995). Quaternary ammonium compounds inhibit and reduce the attachment of viable salmonella typhimurium to poultry tissues. J. Food. Sci.

[3] Cords BR, Burnett SL, Hilgren J, Finely M, Magnuson J (2005). Sanitizers: Halogens, Surface-Active Agents, and Peroxides. Food Sci. Technol.

[4] Kim JW, Slavik MF, Bender FG (1996). Cetylpyridinium chloride (CPC) treatment on poultry skin to reduce attached salmonella. J. Food. Prot.

[5] Wang WC, Li Y, Slavik MF, Xiong H (1997). Trisodium phosphate and cetylpyridinium chloride spraying on chicken skin to reduce attached salmonella typhimurium. J. Food. Prot.

[6] Food and Drug Administrator’s (FDA) (1998). Draft Dental Plaque Subcommittee Report on Cetylpyridinium Chloride. Center for Drug Evaluation and Research.

[7] Renton-Harper P, Addy M, Moran J, Doherty FM, Newcombe RG (1996). A comparison of chlorhexidine, cetylpyridinium chloride, triclosan, and C31G mouthrinse producucts for plaque inhibition. J. Periodontol.

[8] Wilson M, Patel H, Fletcher J (1996). Susceptibility of biofilms of streptococcus sanguis to chlorhexidine gluconate and cetylpyridinium chloride. Oral Microbiol. Immunol.

[9] Mohamed GG, Ali TA, El-Shahat MF, Al-Sabagh AM, Migahed MA, Khaled E (2010). Potentiometric determination of cetylpyridinium chloride using a new type of screen-printed ion selective electrodes. Anal. Chim. Acta.

[10] Mostafa GA (2001). PVC matrix membrane sensor for potentiometric determination of cetylpyridinium chloride. Anal. Sci.

[11] Gerlache M, Kauffmann JM, Quarin G, Vire JC, Bryant GA, Talbot JM (1996). Electrochemical analysis of surfactants. Talanta.

[12] Binder H, Krainer W, Lindner W (1986). Reaction gas-chromatographic determination of cetylpyridinium chloride. Arch. Pharm. (Weinheim, Ger.).

[13] Wang J, Lu J, Zhang L, Hu Y (2003). Determination of cetylpyridinium chloride and tetracaine hydrochloride in buccal tablets by RP-HPLC. J. Pharm. Biomed. Anal.

[14] Heinig K, Vogt C (1999). Determination of surfactants by capillary electrophoresis. Electrophoresis.

[15] Oztekin N, Erim FB (2005). Determination of cationic surfactants as the preservatives in an oral solution and a cosmetic product by capillary electrophoresis. J. Pharm. Biomed. Anal.

[16] Safavi A, Karimi MA (2002). Flow injection determination of cationic surfactants by using N-bromosuccinimide and N-chlorosuccinimide as new oxidizing agents for luminol chemiluminescence. Anal. Chim. Acta.

[17] Benamor M, Aguersif N, Draa MT (2001). Spectrophotometric determination of cetylpyridinium chloride in pharmaceutical products. J. Pharm. Biomed. Anal.

[18] Helena L, Montes C, Cassella RJ (2010). Reversed flow injection system for the spectrophotometric determination of cetylpyridinium chloride in pharmaceutical products with eriochrome black T in Triton X-100 medium. J. Flow. Injection Anal.

[19] Parham H, Pourreza N, Moradi D (2011). Development of flotation spectrophotometric method for determination of cetylpyridinium chloride in phamaceutical products. Quim. Nova.

[20] Zarapkar SS, Rele RV, Shah SVM (1987). Simple extractive colorimetric determination of cetylpyridinium chloride from pharmaceutical preparation. Indian Drugs.

[21] Benamor M, Aguersif N, Draa MT (2001). Spectrophotometric determination of cetylpyridinium chloride in pharmaceutical products. J. Pharm. Biomed. Anal.

[22] Afkhami A, Nematollahi D, Madrakian T, Hajihadi M (2011). Spectrophotometric determination of cationic surfactants based on their effect on the complexes of chrome azurol S with Be2+ and Al3+ cations. Clean-Soil, Air, Water.

[23] Paleologos EK, Giokas DL, Karayannis MI (2005). Micelle-mediated separation and cloud-point extraction. TrAC-Trend. Anal. Chem.

[24] Liang R, Wang Z, Xu J, Li W, Qi H (2009). Novel polyethylene glycol induced cloud point system for extraction and back-extraction of organic compounds. Sep. Purif. Technol.

[25] Purkair MK, Banerjee S, Mewara S, DasGupta S, De S (2005). Cloud point extraction of toxic eosin dyes using Triton-100 as nonionic surfactant. Water Res.

[26] Zarei AR (2007). Cloud point formation based on mixed micelle in the presence of electrolyte for extraction, preconcentration, and spectrophotometric determination of trace amounts of hydrazine in water and biological samples. Anal. Biochem.

[27] Pourreza N, Zareian M (2009). Determination of orange II in food samples after cloud point extraction using mixed micelles. J. Hazard. Mater.

[28] Silva EL, Rolden PS (2009). Simultaneous flow injection preconcentration of lead and cadmium using cloud point extraction and determination by atomic absorption spectrometry. J. Hazard. Mater.

[29] Silva MF, Fernandez LP, Olsina RA (1998). Monitoring the elimination of gadolinium-based pharmaceuticals, Cloud point preconcentration and spectrophotometric determination of Gd(III)-2-(3,5-dichloro-2-pyridylazo)-5-dimethylaminophenol in urine. Analyst.

[30] Filik H, Sener I, Cekic SD, Kiliç E, Apak Rl (2006). Spectrophotometric determination of paracetamol in urine with tetrahydroxycalix[4] arene as a coupling reagent and preconcentration with Triton X-114 using cloud point extraction. Chem. Pharm. Bull.

[31] Sun C, Xie Y, Tian Q, Liu H (2008). Analysis of glycyrrhizic acid and liquiritin in liquorice root with microwave-assisted micellar extraction and pre-concentration. Phytochem. Anal.

[32] Madej K (2009). Microwave-assisted and cloud-point extraction in determination of drugs and other bioactive compounds. TrAC-Trend. Anal. Chem.

[33] Bavili Tabrizi A, Harasi M (2012). Applying cloud point extraction technique for the extraction of oxazepam from human urine as a colour or fluorescent derivative prior to spectroscopic analysis methods. Drug Test. Anal.

[34] Ingle JD, Crouch JR (1988). Spectrochemical Analysis.

[35] Afkhami A, Madrakian T, Siampour H (2006). Highly selective determination of trace quantities of mercury in water samples after preconcentration by the cloud point extraction method. Intern. J. Environ. Anal. Chem.

[36] Miller JC, Miller JN (2000). Stasitical for Analytical Chemistry.

